# Thanks to reviewers

**DOI:** 10.1007/s40037-014-0153-9

**Published:** 2014-12-19

**The Editorial Team would like to extend our gratitude to all reviewers for their time and advice that helped us to assess and improve the papers that were submitted to us in 2014. The reviewers’ critical and constructive feedback greatly helped to raise the journal’s quality.**

Azam Afzalm, Meenakshi Akhilesh, Arunodaya Barman, Esther Bergman, Shivayogi Bhusnurmath, A.B. Bijnen, Sanneke Bolhuis, B. Bonke, Nicole Borges, Jan Borleffs, Joseph Donald Boudreau, Johan Bredée, Jacqueline Broerse, Jamiu Busari, Diana Callender, Richard Cherry, Jeffrey Cheung, Jennifer Cleland, Lara Cooke, Eugene Custers, Mary E.W. Dankbaar, A.Deketelaere, Hanke Dekker, Marja Dijksterhuis, Nisha Dogra, G. Dom, Jeroen Donkers, Tim Dornan, Jos Draaisma, Robbert Duvivier, Liz Farmer, Dario Fernandes, C.R.M.G. Fluit, Janneke Frambach, Joost Frenkel, Mark Goldszmidt, Esther Groot, F.J.M. Grosfeld, Sigrid Harendza, Imad Hassan, Richard Hays, Esther Helmich, Marcus Henning, Carol Herbert, Elspeth Hill, Sören Huwendiek, George Ibrahim, Lamis Kaddam, Ellen Kok, A.W.M. Kramer, W.D.J. Kremer, Frank Krings, Kulamakan Kulasegaram, Rashmi Kusurkar, Vicki Leblanc, Janet Lefroy, Joule Li, Lorelei Lingard, Mario Maas, Karen Mann, Stephen Manuel, Nicole Mastenbroek, Robert McKinley, Sonya Miller, Sandra Monteiro, Friso Muntinghe, Kichu Nair, Laura Naismith, Gita Negi, Zineb Nouns, Arko Oderwald, Maria Opfermann, Helen O'Sullivan, Karlijn Overeem, Elise Paradis, Simon Parson, G. Peeraer, Jan Pols, Shridhar Rawal, Simon Riley, G.M. Rommers, Mirabelle Schaub-de Jong, L.W.T. Schuwirth, Lesley Southgate, Renee Stalmeijer, Lorette Stammen, E.W.M.T. Ter Braak, Pim Teunissen, Rene Tio, Martin Tolsgaard, Andre Tricot, Anke Trigt, Sjoukje Van den Broek, G. Van Hal, Evelyn van Weel-Baumgarten, Marta van Zanten, Mayke Vereijken, Kieran Walsh, Michiel Westerman, Amy Wilson-Delfosse, Kalman Winston, Yolande Witman, Nicole Woods, Barbara Workman, Sarah Wright, Raniai Zaini

